# NF-*κ*B-dependent and -independent epigenetic modulation using the novel anti-cancer agent DMAPT

**DOI:** 10.1038/cddis.2014.569

**Published:** 2015-01-22

**Authors:** H Nakshatri, H N Appaiah, M Anjanappa, D Gilley, H Tanaka, S Badve, P A Crooks, W Mathews, C Sweeney, P Bhat-Nakshatri

**Affiliations:** 1 Department of Surgery, Indiana University School of Medicine, Indianapolis, IN, USA; 2 Department of Biochemistry and Molecular Biology, Indiana University School of Medicine, Indianapolis, IN, USA; 3 Department of Medical and Molecular Genetics, Indiana University School of Medicine, Indianapolis, IN, USA; 4 Department of Pathology and Laboratory Medicine, Indiana University School of Medicine, Indianapolis, IN, USA; 5 Department of Pharmaceutical Sciences, University of Arkansas, Little Rock, AR, USA; 6 Leuchemix, Inc., Woodside, CA, USA; 7 Lank Center for Genitourinary Oncology, Dana-Farber Cancer Institute, Boston, MA, USA

## Abstract

The transcription factor nuclear factor-kappaB (NF-*κ*B) is constitutively active in several cancers and is a target of therapeutic development. We recently developed dimethylaminoparthenolide (DMAPT), a clinical grade water-soluble analog of parthenolide, as a potent inhibitor of NF-*κ*B and demonstrated *in vitro* and *in vivo* anti-tumor activities in multiple cancers. In this study, we show DMAPT is an epigenetic modulator functioning in an NF-*κ*B-dependent and -independent manner. DMAPT-mediated NF-*κ*B inhibition resulted in elevated histone H3K36 trimethylation (H3K36me3), which could be recapitulated through genetic ablation of the p65 subunit of NF-*κ*B or inhibitor-of-kappaB alpha super-repressor overexpression. DMAPT treatment and p65 ablation increased the levels of H3K36 trimethylases NSD1 (KMT3B) and SETD2 (KMT3A), suggesting that NF-*κ*B directly represses their expression and that lower H3K36me3 is an epigenetic marker of constitutive NF-*κ*B activity. Overexpression of a constitutively active p65 subunit of NF-*κ*B reduced NSD1 and H3K36me3 levels. NSD1 is essential for DMAPT-induced expression of pro-apoptotic BIM, indicating a functional link between epigenetic modification and gene expression. Interestingly, we observed enhanced H4K20 trimethylation and induction of H4K20 trimethylase KMT5C in DMAPT-treated cells independent of NF-*κ*B inhibition. These results add KMT5C to the list NF-*κ*B-independent epigenetic targets of parthenolide, which include previously described histone deacetylase 1 (HDAC-1) and DNA methyltransferase 1. As NSD1 and SETD2 are known tumor suppressors and loss of H4K20 trimethylation is an early event in cancer progression, which contributes to genomic instability, we propose DMAPT as a potent pharmacologic agent that can reverse NF-*κ*B-dependent and -independent cancer-specific epigenetic abnormalities.

Epigenetics is defined as heritable changes in gene expression mediated mostly by DNA methylation and histone tail modifications without changes in DNA sequence.^1^ Epigenetic abnormalities in cancer lead to reprogramming of gene expression resembling embryonic stem cells, loss of tumor suppressors, or reactivation of oncofetal genes.^2, 3^ For example, enhanced histone 3 lysine 27 trimethylation (H3K27me3), mediated by a complex of proteins, including the histone methyltransferase EZH2, causes the silencing of tumor-suppressor genes.^4, 5^

Similar to H3K27me3, histone 3 lysine 36 trimethylation (H3K36me3) has a diverse role in chromatin structure and function.^6^ H3K36me3 regulates transcription of active euchromatin, alternative splicing, DNA repair, and transmission of the memory of gene expression from parents to offspring.^7^ At least eight enzymes methylate H3K36. Nuclear receptor-binding SET domain protein 1 (NSD1; lysine methyltransferases 3B (KMT3B)) and NSD2 are involved in monomethylation and dimethylation, whereas SET domain containing 2 (SETD2; KMT3A) trimethylates H3K36.^6^ However, depletion of NSD1 in certain cells leads to loss of H3K36me3 and altered gene expression.^8, 9^

NSD1 functions as a tumor suppressor in several cancers. For example, epigenetic silencing of NSD1 is observed in neuroblastoma.^9^ NSD1 mutation/deletion is observed in bladder cancer.^10^ NSD1 is one of the genes essential for anti-estrogen sensitivity in breast cancer.^11^ NSD1 is also described as an oncogene in acute myeloid leukemia in which a chromosomal translocation creates nucleoporin 98 kDa (NUP98):NSD1 fusion protein, and the expression is driven by NUP98 regulatory elements.^12^ NSD2 is an oncogene, whereas SETD2 is inactivated through mutation/deletion in multiple cancers.^10, 13, 14^ Interestingly, H3K36me3 and H3K27me3 on a gene may be mutually exclusive, suggesting distinct role of modulators of these modifications on gene expression.^15^

Loss of histone 4 lysine 20 trimethylation (H4K20me3) is a hallmark of cancer.^16^ The histone methyltransferases KMT5B (SUV4-20H1) and KMT5C (SUV4-20H2) mediate histone H4K20me3, whereas PHD finger protein 2 (PHF2) demethylates this residue.^17, 18^ NSD1 is also reported to trimethylate H4K20.^9^ H4K20me3 is necessary for the repressive pathway that induce pericentric heterochromatin required for G2/M arrest in response to DNA damage and recruitment of DNA repair complex.^17, 19^ In addition, loss of H4K20me3 is associated with telomere elongation and derepression of telomere recombination.^20^ Depletion of KMT5B or KMT5C leads to increased telomere elongation, which is common in cancer.^20^

Developing drugs that reverse cancer-specific histone modifications is one of our goals. Towards this end, we targeted the transcription factor nuclear factor-kappaB (NF-*κ*B), which is activated in cancers and regulates inflammation-associated epigenetic changes.^21, 22^ Using pharmacological and genomic approaches, we show upregulation of NSD1 and SETD2 upon NF-*κ*B inhibition. Dimethylaminoparthenolide (DMAPT), the pharmacological inhibitor of NF-*κ*B, additionally increased KMT5C and H4K20me3 independent of its NF-*κ*B inhibition attribute.

## Results

### The effects of DMAPT on histone modifications

**Figure 1:**
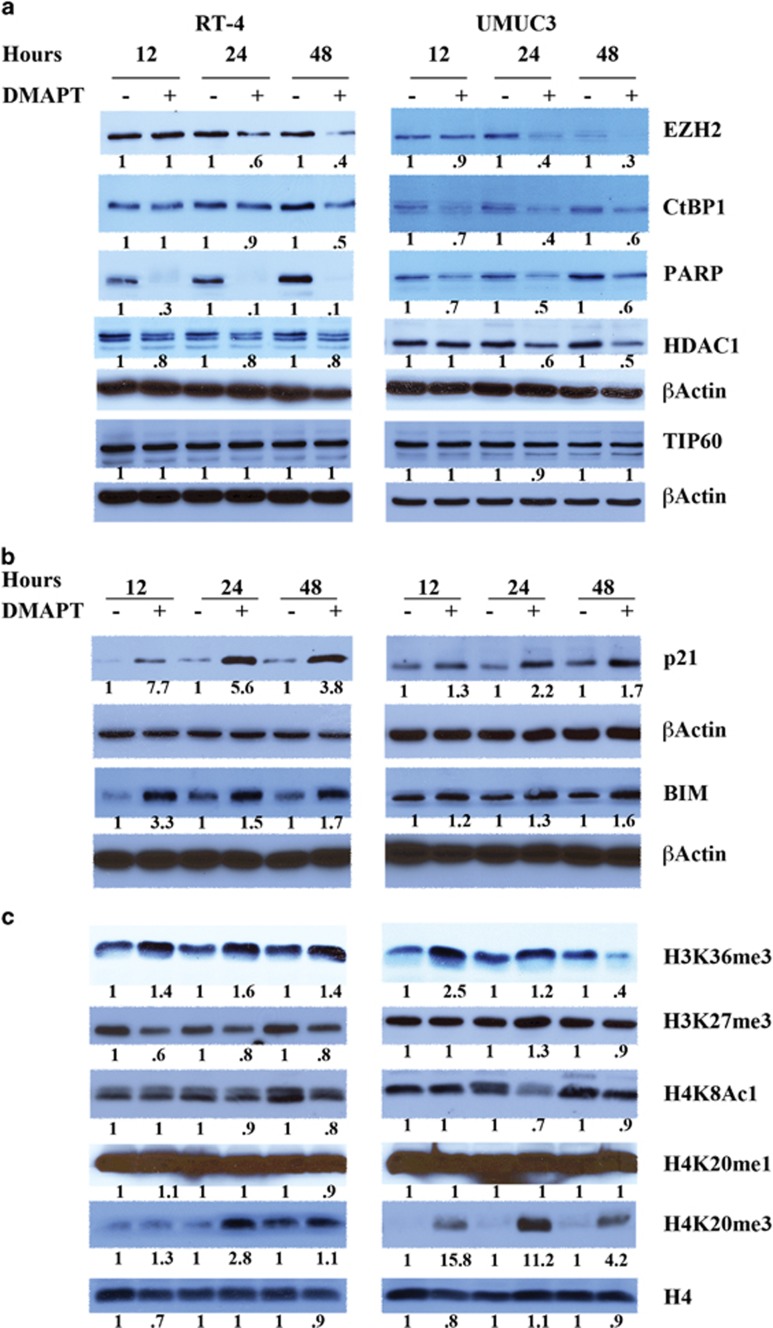
The effect of DMAPT on the expression of epigenetic regulators and on histones. (**a**) DMAPT reduced the levels of EZH2, HDAC-1, CtBP1, and PARP1 in a cell type-dependent manner. Cells were treated with 10 *μ*M DMAPT for the indicated time, and cell lysates were subjected to western blotting. Representative data from two or more experiments are shown. (**b**) DMAPT induced the expression of p21 and BIM. (**c**) The effect of DMAPT treatment on histone modifications. H3K36me3 blots were reprobed with histone H4 as a quality control

Parthenolide, an NF-*κ*B inhibitor, reduces histone deacetylase 1 (HDAC-1) and DNA methyltransferase 1 independent of NF-*κ*B inhibition.^23, 24^ However, it is unclear whether parthenolide alters NF-*κ*B-dependent epigenetic changes that are observed in inflammation.^22^ To test this possibility, we examined the effect of dimethylaminoparthenolide (DMAPT), a water-soluble analog of parthenolide,^25^ on the expression of histone-modifying enzymes and on histone modifications. The study was initiated in bladder cancer cell lines UMUC-3 and RT-4 because of our previous report showing DMAPT-mediated cell cycle arrest and apoptosis of UMUC-3 cells *in vitro* and inhibition of tumor growth in xenograft models.^25^ DMAPT reduced the levels of HDAC-1 in UMUC-3 cells but had a modest effect in RT-4 cells (Figure 1a). DMAPT reduced EZH2 and C-terminal-binding protein 1 (CtBP1) more efficiently in UMUC-3 cells compared with RT-4 cells. In contrast, the effect of DMAPT on poly-(ADP-ribose) polymerase (PARP)-1 was more pronounced in RT-4 cells. These changes in histone-modifying enzyme levels correlated with an increase in the levels of p21 and pro-apoptotic BIM (Figure 1b). It is unlikely that the effect of DMAPT on the above proteins is due to genotoxic stress/DNA-damage response, because DMAPT did not alter TIP60 (histone acetyltransferase Tip60) levels, which is typically activated upon double-stranded DNA break^26^ (Figure 1a).

We next determined the effects of DMAPT on histone H3 and H4 modifications. DMAPT increased the levels of H3K36me3 by 1.6–2.5-fold, although the induction was transient in UMUC-3 cells (Figure 1c). Increase in H3K36me3 was accompanied with decrease in H3K27me3 in RT-4 cells only, despite DMAPT reducing EZH2 levels in both cell types. Overall, DMAPT displayed higher effect on epigenetic machinery in RT-4 cells compared with UMUC-3 cells.

With respect to histone H4, DMAPT increased H4K20me3 by 2.8–16-fold (Figure 1c). H4K8 acetylation was modestly lower in DMAPT-treated cells, although this change was observed only after 24 h of treatment. H4K8 acetylation is usually an inflammatory response mediated by NF-*κ*B, and the effect of DMAPT on this acetylation is consistent with its ability to inhibit NF-*κ*B.^27^ Among various histone modifications examined, H3K36me3 and H4K20me3 were consistently elevated in DMAPT-treated cells.

### NF-*κ*B inhibition through genomic approaches results in elevated H3K36me3

**Figure 2:**
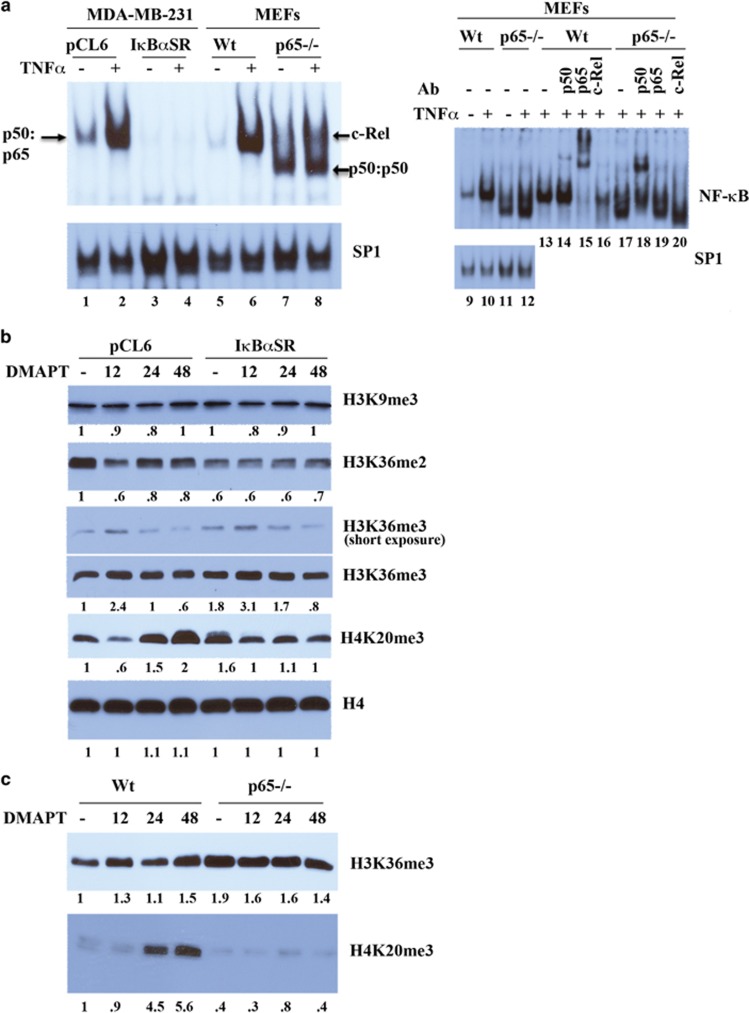
The role of NF-*κ*B in DMAPT-mediated histone modifications. (**a**) NF-*κ*B DNA-binding activity in MDA-MB-231 with or without I*κ*B*α*SR overexpression and in MEFs with and without p65 deletion. Cells were incubated with or without TNF*α* for 15 min, and NF-*κ*B and SP-1 (as a control) DNA-binding activity was measured by electrophoretic mobility gel shift assay. Antibody supershift assay with extracts from wild-type and p65−/− cells is shown in the right side (lanes 13–20). (**b**) The effect of I*κ*B*α*SR overexpression on DMAPT-induced histone modification in MDA-MB-231 cells. Basal H3K36me3 was higher in cells with I*κ*B*α*SR overexpression compared with parental cells with empty lentivirus (pCL6), and it was further increased by DMAPT. (**c**) P65−/− MEFs showed elevated basal H3K36me3 but not H4K20me3 compared with wild-type MEFs. DMAPT increased H3K36me3 and H4K20me3 in wild-type MEFs

We used two model systems to determine which among the activities of DMAPT described above can be recapitulated through genomic inhibition of NF-*κ*B. The first model system involved overexpression of inhibitor-of-kappaB alpha super-repressor (I*κ*B*α*SR), and the second approach utilized mouse embryonic fibroblasts (MEFs) lacking the p65 subunit of NF-*κ*B.^28, 29^ Unfortunately, UMUC-3 cells overexpressing I*κ*B*α*SR could not be maintained for a prolonged time. We had previously demonstrated feasibility of generating MDA-MB-231 breast cancer cells overexpressing I*κ*B*α*SR.^28^ MDA-MB-231 cells have constitutively active NF-*κ*B, and I*κ*B*α*SR reduced basal and tumor necrosis factor alpha (TNF*α*)-inducible activation of NF-*κ*B (Figure 2a). P65−/− MEFs lack p65 DNA-binding activity but displayed p50 and c-Rel DNA-binding activity as evident from antibody supershift assay (Figure 2a, compare lanes 6 with 8, 15 with 19, and 12 with 20).

Basal H3K36me3 was 1.8-fold higher in I*κ*B*α*SR cells compared with parental cells, which coincided with lower H3K36 dimethylation (Figure 2b). These results suggest that NF-*κ*B inhibition through genomic approaches can recapitulate the effects of DMAPT on H3K36me3. The effect of DMAPT on H4K20me3 in MDA-MB-231 cells was not as robust as in UMUC-3 cells, suggesting cell type specificity (Figure 2b).

We next examined wild-type and p65−/− MEFs for histone modifications. Basal H3K36me3 was 2-fold higher in p65−/− cells compared with wild-type cells (Figure 2c). Interestingly, DMAPT significantly increased H4K20me3 in wild-type but not in p65−/− cells. In addition, basal H4K20me3 levels were the same in wild-type and p65−/− cells. Reasons for this discrepancy are unknown.

### DMAPT treatment or NF-*κ*B inhibition results in elevated NSD1 and SETD2

**Figure 3:**
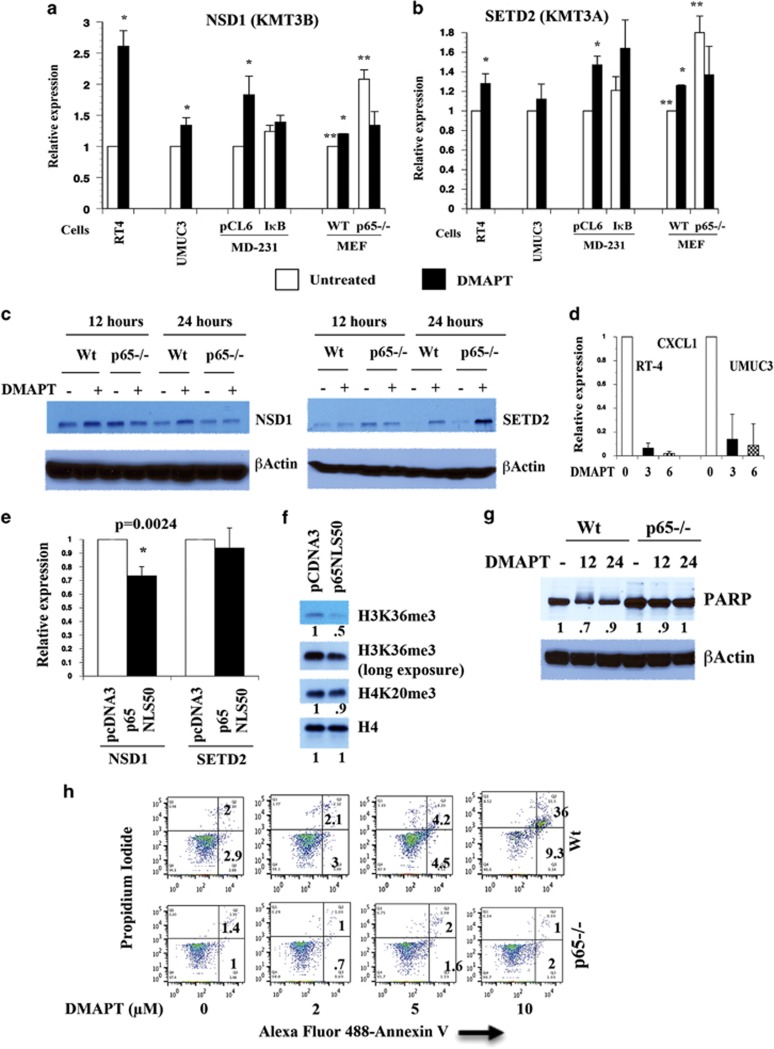
DMAPT induced histone H3K36 methyltransferases NSD1 and SETD2. (**a**) The effect of DMAPT on NSD1 expression. Cells were treated with 10 *μ*M DMAPT for 12 h, and qRT-PCR was performed to measure NSD1 mRNA. Average±S.E.M. from biological replicates is shown. Asterisk in this and subsequent figures denotes statistically significant differences with a *P*-value of <0.05. (**b**) The effect of DMAPT on SETD2 expression. Basal SETD2 level was significantly higher in p65−/− cells compared with wild-type cells. (**c**) The effect of DMAPT on NSD1 and SETD2 proteins in wild-type and p65−/− MEFs. (**d**) DMAPT reduced CXCL1 mRNA in RT-4 and UMUC-3 cells. (**e**) The effect of p65NLS50 expression on NSD1 and SETD2 expression in p65−/− cells. Cells were transfected with empty vector pcDNA3 or p65NLS50 vector (10 *μ*gs), and RNA was analyzed for NSD1 and SETD2 levels 48 h after transfection. (**f**) H3K36me3 and H4K20me3 levels in pcDNA3- and p65NLS50-transfected cells. Experiments were done as in panel (**e**). (**g**) DMAPT (10 *μ*M) reduced PARP-1 in wild-type but not p65−/− cells. (**h**) Wild-type but not p65−/− cells were sensitive to DMAPT-induced apoptosis. Cells were treated with the indicated concentration of DMAPT for 24 h, and apoptosis was measured using Annexin V labeling. Representative results are shown

To determine the targets of DMAPT that are responsible for histone H3K36me3, we measured the levels of NSD1, NSD2, and SETD2. DMAPT increased the expression of NSD1 in both RT-4 and UMUC-3 cells (Figure 3a). Highest induction was observed in RT-4 cells (2.6-fold), which also showed persistent elevation of H3K36me3 upon DMAPT treatment.

To obtain definitive evidence for the negative regulation of NSD1 expression by NF-*κ*B, we measured NSD1 in MDA-MB-231 cells overexpressing I*κ*B*α*SR and p65−/− MEFs. DMAPT increased NSD1 in MDA-MB-231 and wild-type MEFs although the level of induction was not as high as in RT-4 cells (Figure 3a). Basal expression of NSD1 was 2.1-fold higher in p65−/− cells compared with wild-type cells confirming that inhibition of canonical NF-*κ*B results in elevated NSD1. DMAPT treatment or I*κ*B*α*SR overexpression had negligible effect on NSD2 levels (Supplementary Figure S1A).

Similar to NSD1, SETD2 was responsive to DMAPT treatment or NF-*κ*B inhibition through genomic approaches, although the degree of response was lower and was cell type-specific (Figure 3b). Critically, basal SETD2 levels were higher in p65−/− cells (~2-fold) compared with wild-type cells, demonstrating an effect of NF-*κ*B on its expression.

To ensure that DMAPT treatment or NF-*κ*B inhibition leads to elevated NSD1 and SETD2 proteins, western blotting was performed. One set of antibodies among many tested recognized proteins of expected size (~300 kDa) and were reactive against only murine proteins. Basal NSD1 and SETD2 levels were higher in untreated p65−/− MEFs compared with wild-type MEFs (Figure 3c). DMAPT increased NSD1 protein in wild-type MEFs. In contrast, SETD2 protein levels were higher in DMAPT-treated wild-type and p65−/− cells. It appears that cell confluency impacts basal NSD1 and SETD2 levels, as the basal expression was lower in untreated cells plated for 24 h compared with cells plated for 12 h.

Although NF-*κ*B predominantly increases the expression of genes, it represses few genes,^30, 31, 32^ and this study adds NSD1 and SETD2 to the list of NF-*κ*B repressed genes. To ensure that induction of NSD1 and SETD2 by DMAPT is not due to a global positive effect of DMAPT on transcription, we measured the effect of DMAPT on the expression of NF-*κ*B-inducible CXCL1.^33^ DMAPT reduced the level of CXCL1 in both UMUC-3 and RT-4 cells (Figure 3d), confirming the specificity in the effects of DMAPT on NSD1 and SETD2.

To further demonstrate the negative relationship between NF-*κ*B and NSD1, we transfected p65−/− cells with a constitutively active p65 subunit of NF-*κ*B in which nuclear localization signal of p65 has been replaced with that of p50 subunit (p65NLS50).^34^ P65−/− cells transfected with p65NLS50 showed lower levels of NSD1 but not SETD2 (Figure 3e). The reason for the lack of effects of p65NLS50 on SETD2 is unknown. H3K36me3 but not H4K20Me3 levels were lower in p65NLS50-transfected cells compared with the vector-transfected cells (Figure 3f), demonstrating the existence of an NF-*κ*B:NSD1:H3K36me3 axis.

To correlate the effect of DMAPT on NSD1 and H3K36me3 on cellular phenotype, we subjected untreated and DMAPT-treated wild-type and p65−/− MEFs to western blotting analysis for PARP-1 and apoptosis assay using Annexin V labeling. DMAPT reduced the levels of PARP-1 (Figure 3g) and induced apoptosis (Figure 3h) in wild-type but not p65−/− cells. Resistance of p65−/− cells to DMAPT-induced apoptosis is surprising, considering that these cells have higher basal levels of NSD1. One possibility is that, in the absence of p65, cells adapt to alternative survival mode, and inducible/acute suppression of NF-*κ*B activity is required to evoke apoptotic response. We could not efficiently reduce NSD1 in these cells by siRNA owing to poor transfection efficiency, which prevented us from confirming the role of DMAPT-induced NSD1 in apoptosis.

### DMAPT increases the expression of KMT5C (SUV4-20H2) in a cell type-dependent manner

**Figure 4:**
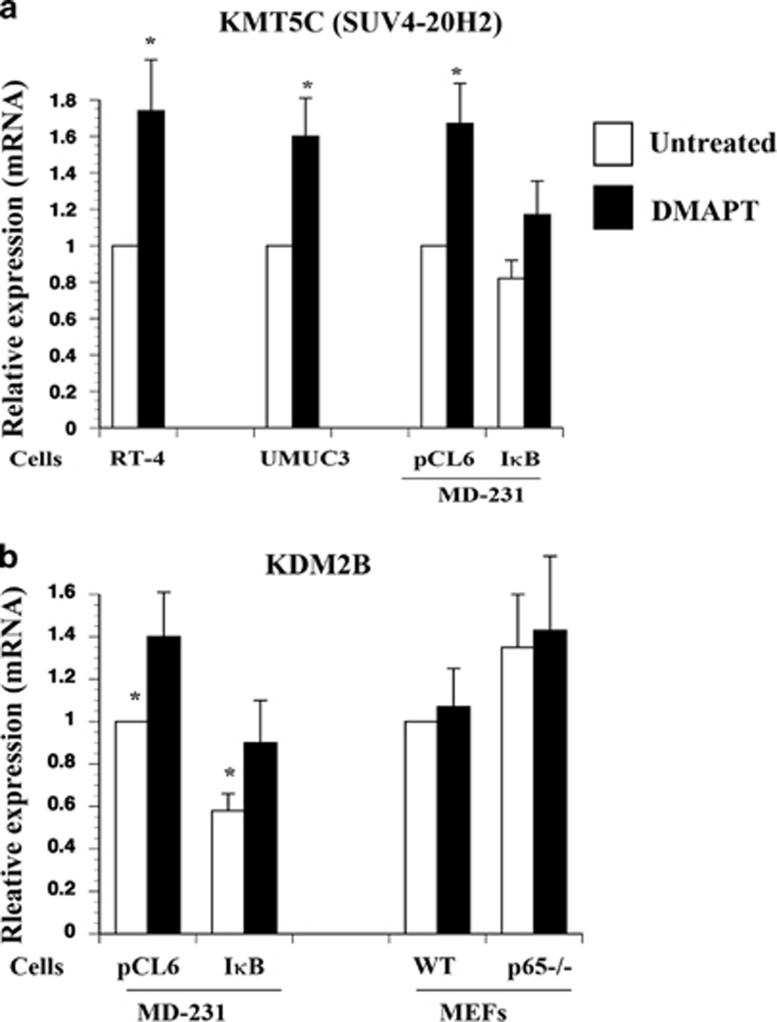
DMAPT increased histone H4K20 trimethyltransferase KMT5C in a cell type-dependent but NF-*κ*B-independent manner. (**a**) KMT5C expression in DMAPT-treated cells. Cells were treated with DMAPT for 6 h. Note that I*κ*B*α*SR overexpression had minimum effect on KMT5C expression in MDA-MB-231 cells. (**b**) NF-*κ*B activity is required for KDM2B expression in MDA-MB-231 but not in MEFs. I*κ*B*α*SR overexpression caused significant drop in basal KDM2B levels in MD-MB-231 cells, whereas p65 knockout did not have an effect on its expression in MEFs

As noted in Figure 1, DMAPT increased H4K20me3 although this induction appeared to be NF-*κ*B-independent, because p65−/− cells did not display elevated levels of H4K20me3 (Figure 2). KMT5C/SUV4-20H2 is one of the methyltransferases involved in this trimethylation.^35^ DMAPT increased the expression of KMT5C consistently in RT-4, UMUC-3, and MDA-MB-231 cells (Figure 4a). However, there was marked experimental variability in the effect of DMAPT on KMT5C expression in MEFs (induction in two out of the three experiments in wild-type but not p65−/− MEFs, Supplementary Figure S1B). Moreover, I*κ*B*α*SR overexpression in MDA-MB-231 cells did not lead to elevated KMT5C expression. Based on these results, we conclude that DMAPT induces KMT5C in a cell type-dependent but NF-*κ*B-independent manner. In this regard, DMAPT induces c-Jun N-terminal kinase (JNK) and generates of reactive oxygen species independent of NF-*κ*B inhibition.^25^

### H3K36 demethylases are not the targets of DMAPT

Previous studies have demonstrated that lysine demethylases 2A (KDM2A; FBXL11) and KDM2B (FBXL10) demethylate monomethylated and dimethylated H3K36, whereas KDM4A, KDM4B, KDM4C, KDM4D, and NO66 demethylate H3K36 dimethyl and trimethyl moieties.^36^ Additionally, independent studies have demonstrated that NSD1, NF-*κ*B, and KDM2A form a feedback axis in which NSD1 methylates p65 subunit and activates NF-*κ*B, which leads to elevated expression of KDM2A.^37^ KDM2A in turn demethylates and inactivates NF-*κ*B. KDM2B is also an NF-*κ*B-inducible gene.^38^ As NF-*κ*B inhibited NSD1 expression, we next examined the effect of DMAPT or I*κ*B*α*SR overexpression on the expression of KDMs associated with demethylation of H3K36me3. KDM2B showed a distinct pattern of expression upon pharmacological or genetic inhibition of NF-*κ*B (Figure 4b). DMAPT treatment did not alter KDM2B in MDA-MB-231 cells. However, I*κ*B*α*SR overexpression reduced its basal expression consistent with reported NF-*κ*B-inducible expression of this gene.^38^ Interestingly, wild-type and p65−/− MEFs showed similar levels of KDM2B and DMAPT had no effect on its expression, which suggests cell type-specific requirement of NF-*κ*B for its expression. Lack of effect of DMAPT on its expression is intriguing, which could be due to competing signaling pathways activated/repressed by DMAPT compensating for the loss of NF-*κ*B activity. In this respect, we had previously demonstrated the ability of DMAPT or parthenolide to induce JNK independent of NF-*κ*B inhibition.^25, 39^ Note that I*κ*B*α*SR overexpression did not have an effect on KDM4A and KDM4B expression, although DMAPT modestly increased their expression, which could be an off-target effect or a non-specific response of cells to drug treatment (Supplementary Figure S1C). Overall, these results favor the possibility that increase in KMTs rather than reduced levels of KDMs contribute to H3K36 trimethylation in DMAPT-treated cells. The effect of DMAPT treatment, I*κ*B*α*SR overexpression, or p65 deletion on epigenetic markers and the expression of epigenetic modulators are summarized in Table 1.

### NSD1 is essential for DMAPT-induced BIM expression

**Figure 5:**
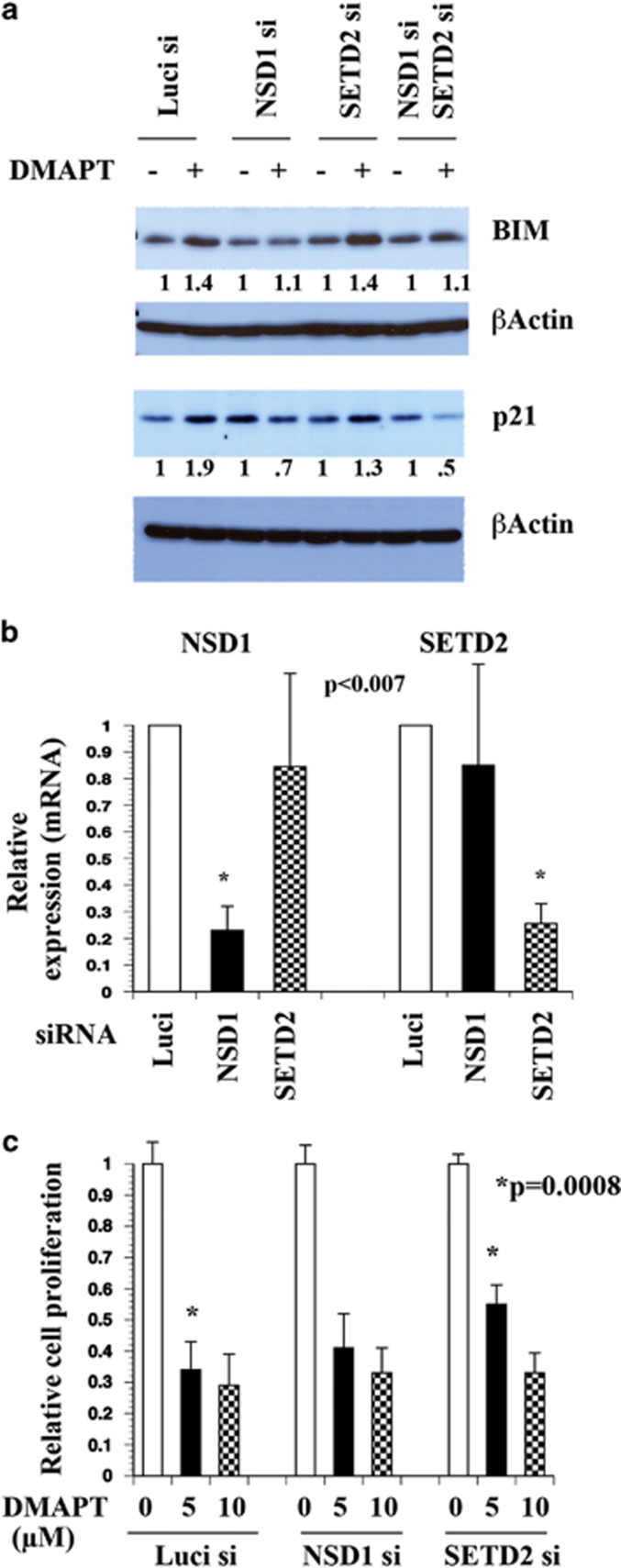
NSD1 is essential for DMAPT-induced BIM and p21 expression. (**a**) BIM and p21 levels in untreated and DMAPT-treated UMUC-3 cells additionally transfected with siRNA against luciferase as a negative control, NSD1, SETD2, or both NSD1 and SETD2. Cells were treated after 3 days of siRNA transfection with DMAPT and harvested for proteins after 24 h of DMAPT treatment. NSD1 siRNA prevented DMAPT-induced BIM and p21. For unknown reasons, basal p21 levels in NSD1 siRNA-treated cells showed remarkable experimental variability in UMUC-3 cells. Densitometry values show comparison between untreated controls and DMAPT-treated cells with respective controls normalized to 1. (**b**) NSD1 and SETD2 mRNA levels in siRNA-transfected cells. Respective mRNAs were measured 4 days after siRNA transfection. (**c**) SETD2 is required for DMAPT-mediated growth inhibition. Cells were plated in a 96-well plate and treated with DMAPT for 48 h. Cell proliferation was measured using BrDU-incorportation ELISA

To determine whether NSD1 and SETD2 have any role in DMAPT-induced BIM and p21 expression, we examined the effects of siRNA against NSD1 and SETD2 on their expression in UMUC-3 cells. SiRNA against NSD1 but not SETD2 prevented DMAPT-induced BIM and p21 expression (Figure 5a). For unknown reason, we observed elevated basal p21 in NSD1 siRNA-treated cells. Because of the transfection-induced stress, we consistently observed lower p21 and BIM induction by DMAPT under this experimental condition. siRNAs were effective in reducing the levels of target gene mRNAs (Figure 5b). We were unable to reproducibly detect the effects of NSD1 and SETD2 siRNA on basal and/or DMAPT-induced H3K36me3 (except in MCF-7 cells) or H4K20me3.

We next examined whether NSD1 and SETD2 are required for DMAPT-induced growth inhibition. Cells transfected with SETD2 siRNA but not NSD1 siRNA were partially resistant to DMAPT compared with control siRNA-transfected cells (Figure 5c).

### NSD1 and SETD2 expression in bladder and breast cancer

**Figure 6:**
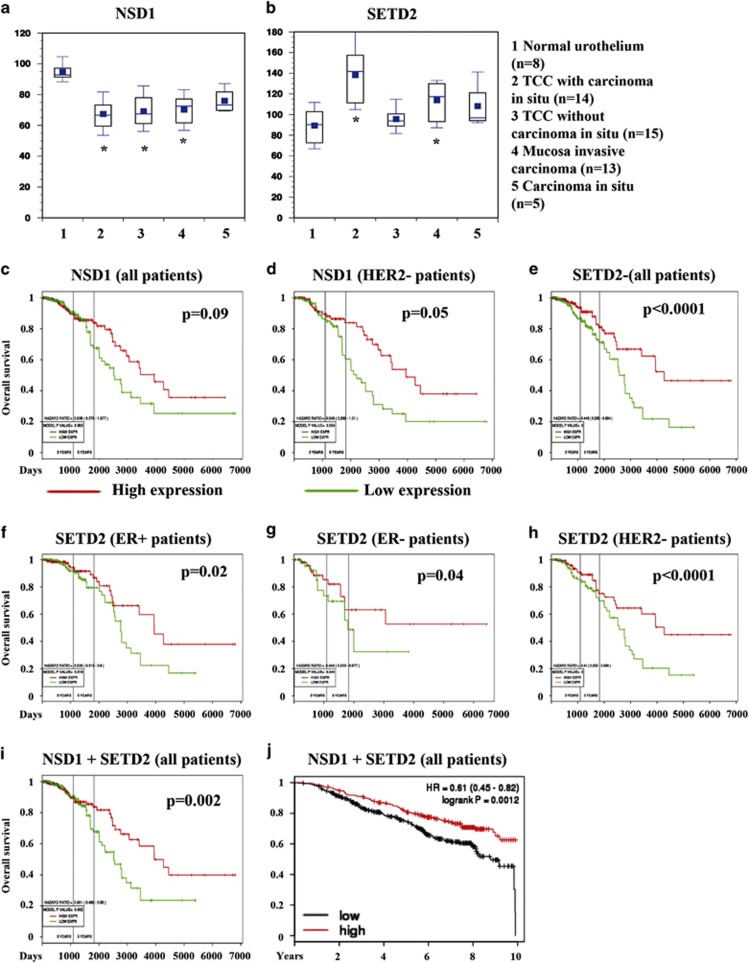
Prognostic value of NSD1 and SETD2 in cancer. (**a**) Levels of NSD1 in normal urothelium and various stages of bladder cancer. NCBI GEO data set GDS1479, which contains NSD1 expression levels (one probe set) in normal urothelium and different stages of bladder cancer, was used to generate this figure. (**b**) Levels of SETD2 in normal urothelium and various stages of bladder cancer. Data were generated using the same data set as in panel (**a**) except that signals were average of three probes that measured SETD2 mRNA. (**c**–**j**) Prognostic value of NSD1, SETD2, or combination in different subtypes of breast cancer. Public databases created by us^43^ (**c**–**i**) and others^44^ (**j**) were used to generate these figures

Analysis of the public database cBioportal revealed significant mutations/deletion of NSD1 and SETD2 in multiple cancers, including bladder cancer (www.cbioportal.org).^40, 41^ In The Cancer Genome Atlas (TCGA) bladder data set (*n*=127), 8% of cases showed deletions, amplification, and/or mutation of NSD1. One percent of breast cancers showed NSD1 alterations (*n*=482). Mutation, amplification, and/or deletion of SETD2 were observed in 10% of bladder cancer (*n*=127). SETD2 alterations were found in 1% of breast cancer (*n*=482). Across multiple cancers, most of the SETD2 alterations involved mutations and deletions but rarely amplification. To determine whether the expression levels of these two genes have diagnostic/prognostic impact, we queried public gene expression databases. In one of the bladder cancer data set in gene expression omnibus (GEO),^42^ we observed significant reduction in NSD1 expression in non-muscle invasive urothelial cancer with or without carcinoma *in situ* and mucosa invasive carcinoma but not in carcinoma *in situ* compared with normal urothelium (Figure 6a). Oncomine search revealed copy number loss and reduced mRNA levels in various bladder cancer stages (Supplementary Figure S2). In contrast to NSD1, SETD2 expression levels did not show any correlation with disease stage. In fact, its expression was elevated in urothelial cancer with carcinoma *in situ* and mucosa invasive carcinoma but not in carcinoma *in situ* cases or cancer without carcinoma *in situ* compared with normal urothelium (Figure 6b). In addition, Oncomine database search did not reveal bladder cancer tumor stage-specific changes in SETD2 expression. However, comparison of SETD2 expression across several cancers demonstrated lower SETD2 expression in multiple cancers, including bladder cancer (fold changes −2.3 and −3 in two studies) (Supplementary Figure S2).

We recently developed an online tool, which enables investigators to determine the prognostic value of genes in >20 data sets with clinical annotation.^43^ With the TCGA breast cancer data set, higher expression of NSD1 showed a trend toward better overall survival when all tumor subtypes were considered (Figure 6c) and significant survival advantage in HER2-negative patients (135 high and 134 low expression cases, Figure 6d). Elevated SETD2 expression correlated with better overall survival in all the subtypes of breast cancer (Figures 6e–h, ER-positive cases—229 high and 229 low; ER-negative cases—68 high and 67 low; HER-negative cases—135 high and 134 low). Combined NSD1 and SETD2 expression levels correlated with better outcome in the TCGA data set (Figure 6i) and in another public database (247 high and 314 low expression cases, Figure 6j).^44^

## Discussion

In this study, we report the ability of the NF-*κ*B inhibitor DMAPT to elevate the expression of NSD1, SETD2, and KMT5C and to increase H3K36me3 and H4K20me3. Induction of NSD1, SETD2, and H3K36me3 could be recapitulated through genomic approaches that inhibited NF-*κ*B. These results reveal H3K36me3 as an epigenetic biomarker of constitutive NF-*κ*B activation in cancer.

### Impact of NSD1–NF-*κ*B axis on cancer

Several epithelial and hematological malignancies show constitutive NF-*κ*B activation, which could lead to reduction in NSD1 levels.^21^ Although NSD1 has been described as an oncogene in leukemia, this oncogenic function is only in the context of NUP98–NSD1 fusion gene where the fusion protein increases H3K36me3 specifically at the HoxA locus and increases HoxA expression.^12^ NF-*κ*B inhibition in this context is less likely to cause upregulation of NUP98–NSD1, because NUP98 regulatory region drives this fusion gene expression. In neuroblastoma, NF-*κ*B inhibitors could potentially restore the expression of epigenetically inactivated NSD1.^9^

In breast cancer, loss of NSD1 expression is associated with resistance to anti-estrogens.^11^ NF-*κ*B has a significant role in anti-estrogen resistant growth of breast cancer cells and NF-*κ*B inhibitors reverse anti-estrogen resistance, which may involve induction of NSD1.^45, 46, 47^ Restoration of NSD1 expression upon DMAPT treatment could also alter sensitivity to other therapies, because NSD1 is essential for BIM expression (Figure 5). BIM polymorphism and BIM levels in treatment-naive cancers determine sensitivity to receptor kinase inhibitors, particularly EGFR and mutant B-RAF signaling pathway inhibitors, glucocorticoids, and paclitaxel.^48^

NSD1 methylates and activates non-histone targets, including the p65 subunit of NF-*κ*B.^37^ However, NSD1 induction due to NF-*κ*B inhibition is less likely to lead to activation of NF-*κ*B target genes, because NF-*κ*B is functionally inactive due to cytoplasmic retention or degradation. NSD1 functions as a co-activator of nuclear receptors, including retinoic acid receptor.^49^ NF-*κ*B inhibition leading to elevated NSD1 may sensitize cancer cells to retinoic acid-induced differentiation.

### Impact of SETD2 repression by NF-*κ*B on cancer

SETD2 is frequently inactivated through mutation and/or deletion in renal, bladder, and breast cancers.^10, 14, 50^ Lower expression of this gene in breast tumors correlated with poor overall survival (Figure 6). DMAPT could potentially be used to restore the expression of SETD2 in tumors, although such a treatment may not be an option in cases with mutation. Apart from histones, SETD2 interacts with p53 and induces p53-dependent pro-apoptotic genes but represses hdm2, a p53 antagonist.^51^ Therefore NF-*κ*B inhibition in tumor cells with intact p53 may trigger a SETD2- and p53-dependent apoptosis. In this respect, DMAPT was more effective in inducing epigenetic changes in RT-4 cells with wild-type p53 compared with p53-mutant UMUC-3 cells.

### NF-*κ*B-mediated repression of gene expression

This study adds to the list of growing number of genes repressed by NF-*κ*B. We had previously demonstrated NF-*κ*B-mediated repression of GADD153.^30^ NF-*κ*B subunit p65, when unphosphorylated at S276, recruits HDAC3 and represses transcription.^52^ Additionally, NF-*κ*B represses transcription by targeting SP-1 and SP-3 transcription factors.^32, 53^ Regulatory regions of NSD1 and SETD2 contain binding sites for NF-*κ*B and SP-1, respectively. It is possible that unphosphorylated p65 occupies NSD1 regulatory region to repress transcription and DMAPT treatment or deletion of p65 leads to derepression and consequent activation. Similarly, NF-*κ*B may target SP-1 to repress SETD2. As basal levels of both NSD1 and SETD2 were higher in p65−/− cells, it is unlikely that NF-*κ*B undergoes repressor to activator switch in DMAPT-treated cells to cause induction of NSD1 and SETD2. The fact that DMAPT treatment or p65 deletion did not have global effects on histone methyltransferases or demethylases suggests that repression of NSD1 and SETD2 is a specific component of NF-*κ*B-mediated epigenetic changes.

KDM6B, which demethylates H3K27, and PHF2, which demethylates H4K20, and KDM2B, which demethylates H3K36me1, H3K36me2, and H3K4me3, have previously been reported as NF-*κ*B-inducible genes.^18, 22, 38^ Although we observed NF-*κ*B-dependent expression of KDM2B, as its levels were lower in I*κ*B*α*SR-overexpressing cells compared with parental cells, we did not observe an effect of DMAPT on other KDMs tested. There are two possible explanations; one is that NF-*κ*B controls their expression in a cell type-dependent manner, and the second is that NF-*κ*B-independent activities of DMAPT cause compensatory increase in the expression of these demethylases, thus nullifying the effects of NF-*κ*B inhibition. The second possibility appears to be the case with KDM2B. Nonetheless, these observations provide explanation as to why results obtained using pharmacological inhibitors are not always compatible with results of gene ablation studies.

Evaluation of the role of NSD1 and SETD2 in DMAPT-induced BIM and p21 expression and cell death showed discordant results. Although NSD1 was required for DMAPT-induced BIM expression, NSD1 knockdown had minimum affect on growth inhibition by DMAPT. In contrast, SETD2 knockdown had no effect on DMAPT-induced BIM and p21 expression, but cells were partially resistant to DMAPT-mediated growth inhibition. At present, SETD2 downstream targets that mediate DMAPT-induced cell growth inhibition in p53-mutant cells are unknown.^51^ Despite gaps in our understanding of this aspect of DMAPT action, data presented here suggest that NSD1 and SETD2 along with H3K36me3 can be used as biomarkers to measure the therapeutic activity of DMAPT as well as any other drugs that inhibit NF-*κ*B activity.

## Materials and Methods

### Cell lines

UMUC-3 cells and MDA-MB-231 cells were maintained in MEM with 10% fetal bovine serum (FBS). RT-4 cells were maintained in McCoy's 5a medium supplemented with 2 mM L-glutamine and 10% FBS. All human cell lines used in this study have been authenticated using the STR systems for cell line identification (Promega, Madison, WI, USA) by a commercial vendor (www.DNAcenter.com) in August 2012. Wild-type and p65−/− MEFs were maintained in DMEM with 10% FBS and were generous gift from Dr. Alex Hoffman (University of California, San Diego, CA, USA) and have been described.^54^

### Antibodies and western blotting analysis

Antibodies against CtBP1 (cat. no. 07-306), H3K36me3 (cat. no. CS204368), H3K9me3 (cat. no. 07-523), Acetyl H3 (cat. no. 06-599), H3K27me3 (cat. no. CS200603), H4K8 acetyl (cat. no. 06-760-MN), H4K20me1 (cat. no. 07-1570), H4K20me3 (cat. no. 07-463), H4 (cat. no. 07-108) and NSD1 (cat. no. 04-1565) were purchased from Upstate Biotechnology/Millipore (Billerica, MA, USA). Antibodies against EZH2 (cat. no. 4905) and HDAC-1 (cat. no. 2062) were purchased from Cell Signaling Technologies (Danvers, MA, USA). BIM (cat. no. 202000) antibody was purchased from Calbiochem/Millipore (Billerica, MA, USA), whereas p21 (cat. no. sc-756) and PARP-1 (cat. no. sc-7150) antibodies were purchased from Santa Cruz Biotechnologies (Santa Cruz, CA, USA). SETD2 antibody (PA5-34934) was purchased from ThermoFisher Scientific (Waltham, MA, USA). Western blotting was performed as described previously,^39^ and representative western blots from three or more experiments are shown. Although primary antibodies in non-histone blots were incubated in Tris-buffered saline-Tween 20 (TBS-T) with 5% non-fat milk power, histone blots with few of the primary antibodies were incubated in 5% BSA containing TBS-T.

### siRNA transfection

Cells were seeded in MEM plus 10% FBS for 48 h and then transfected with 25 nM of siRNA (Dharmacon, Lafayette, CO, USA) using lipofectamine reagent. Cells were treated after 3 days of siRNA transfection with DMAPT and harvested for proteins or RNA after 24 h of DMAPT treatment.

### Quantitative reverse transcription-PCR (qRT-PCR)

Independent samples of total RNA were prepared using RNAeasy kit (Qiagen, Valencia, CA, USA). First-strand cDNA was synthesized using random hexamers and superscript II reverse transcriptase (Invitrogen, Carlsbad, CA, USA). qRT-PCR was performed using the SYBR green according to the manufacturer's protocol (Applied Biosystems, Foster City, CA, USA). Expression of *β*-actin housekeeping gene was used as an internal control for normalization between samples. Results presented are from three or more experiments, and average±S.E.M. are presented. Primer sequences used for qRT-PCR are provided in Supplementary Table S1.

### Apoptosis assay

Apoptosis was measured using Annexin V labeling using the Apoptosis Assay Kit from Invitrogen, and the number of apoptotic cells after 24 h of DMAPT treatment was measured by flow cytometry. Both floating and adherent cells were collected and stained with Alexa Fluor-488-conjugated Annexin V and propidium iodide. Annexin V-positive cells are apoptotic, propidium iodide-positive cells are necrotic and double-positive cells are necroapoptotic.

### Histone extraction

Cells were lysed with Triton Extraction Buffer (TEB: PBS containing 0.5% Triton X 100(v/v), 2 mM phenylmethylsulfonylfluoride (PMSF), 0.02% (w/v) NaN_3_) and centrifuged at 6500 × *g* for 10 min at 4 °C to collect nuclei. The histones were subsequently extracted with 0.2 M HCl (Abcam histone extraction protocol, Cambridge, MA, USA).

### Electrophoretic mobility gel shift assay

MDA-MB-231 and MEF cells were harvested in their exponential growth phase with or without TNF*α* (5 ng/ml, R&D Systems, Minneapolis, MN, USA) treatment for 15 min and assayed for NF-*κ*B and SP-1 (as a control) DNA-binding activity as described previously.^39^ Antibodies for supershift assays were purchased from Santa Cruz (c-Rel, cat. no. sc-070) and Millipore (p65, cat. no. 06-418; p50, cat. no. 06-886).

### Statistical analysis

Results of qRT-PCR were analyzed using the GraphPad software (www.Graphpad.com). Analysis of variance was used to determine the *P*-values between mean measurements. A *P*-value of *<*0.05 was deemed significant.

### Analysis of public databases for prognostic relevance of NSD1 and SETD2

Expression array data of various bladder cancer stages were obtained from NCBI GEO (GDS1479), and average±S.D. was calculated. NSD1 expression data were from a single affymetrix probe available in the data set, whereas average from three probes was used for SETD2. For breast cancer, analysis of TCGA data set^55^ for NSD1 and SETD2 expression is presented although similar analysis using a public data set with gene expression pattern in tumors of 1809 breast cancer patients yielded similar results.^44^

## Figures and Tables

**Table 1:** Summary of the effects of DMAPT treatment, I*κ*B*α*SR overexpression, and p65 depletion on epigenetic markers and the expression of epigenetic regulators

	DMAPT	I*κ*B*α*SR	P65−/−
H3K36me3	Yes	Yes	Yes
H4K20me3	Yes	No	No
NSD1	Yes	?^a^	Yes
SETD2	Yes	?^a^	Yes
KMT5C	Yes	No	No
KDM2B	No	Yes	No

a Trend of an effect but did not reach statistical significance

## Abbreviations

CtBP1: C-terminal-binding protein 1
DMAPT: dimethylaminoparthenolide
GEO: gene expression omnibus
H3K27me3: histone 3 lysine 27 trimethylation
H3K36me3: histone 3 lysine 36 trimethylation
H4K20me3: histone 4 lysine 20 trimethylation
HDAC-1: histone deacetylase 1
I*κ*B*α*SR: inhibitor-of-kappaB alpha super-repressor
JNK: c-Jun N-terminal kinase
KMT: lysine methyltransferases
KDM: lysine demethylases
MEF: mouse embryonic fibroblast
NF-*κ*B: nuclear factor-kappaB
NSD1: nuclear receptor-binding SET domain protein 1
NUP98: nucleoporin 98 kDa
PARP: poly-(ADP-ribose) polymerase
PHF2: PHD finger protein 2
qRT-PCR: quantitative reverse transcription-PCR
SETD2: SET domain containing 2
TBS-T: Tris-buffered saline-Tween 20
TCGA: The Cancer Genome Atlas
TNF*α*: tumor necrosis factor alpha

## Notes

HN, PAC, WM, and CS are the co-founders of Leuchemix Inc., which is developing parthenolide and its analogs as anti-cancer agents. The other authors declare no conflict of interest.

## References

[bib1] Sharma S, Kelly TK, Jones PA. Epigenetics in cancer. Carcinogenesis 2010; 31: 27–36. 19752007 10.1093/carcin/bgp220 PMC2802667

[bib2] Jones PA, Baylin SB. The epigenomics of cancer. Cell 2007; 128: 683–692. 17320506 10.1016/j.cell.2007.01.029 PMC3894624

[bib3] Ben-Porath I, Thomson MW, Carey VJ, Ge R, Bell GW, Regev A et al. An embryonic stem cell-like gene expression signature in poorly differentiated aggressive human tumors. Nat Genet 2008; 40: 499–507. 18443585 10.1038/ng.127 PMC2912221

[bib4] Kanno R, Janakiraman H, Kanno M. Epigenetic regulator polycomb group protein complexes control cell fate and cancer. Cancer Sci 2008; 99: 1077–1084. 18422744 10.1111/j.1349-7006.2008.00797.x PMC11159164

[bib5] Schuettengruber B, Chourrout D, Vervoort M, Leblanc B, Cavalli G. Genome regulation by polycomb and trithorax proteins. Cell 2007; 128: 735–745. 17320510 10.1016/j.cell.2007.02.009

[bib6] Wagner EJ, Carpenter PB. Understanding the language of Lys36 methylation at histone H3. Nat Rev Mol Cell Biol 2012; 13: 115–126. 22266761 10.1038/nrm3274 PMC3969746

[bib7] Schmidt CK, Jackson SP. On your mark, get SET(D2), go! H3K36me3 primes DNA mismatch repair. Cell 2013; 153: 513–515. 23622237 10.1016/j.cell.2013.04.018

[bib8] Lucio-Eterovic AK, Singh MM, Gardner JE, Veerappan CS, Rice JC, Carpenter PB. Role for the nuclear receptor-binding SET domain protein 1 (NSD1) methyltransferase in coordinating lysine 36 methylation at histone 3 with RNA polymerase II function. Proc Natl Acad Sci USA 2010; 107: 16952–16957. 20837538 10.1073/pnas.1002653107 PMC2947892

[bib9] Berdasco M, Ropero S, Setien F, Fraga MF, Lapunzina P, Losson R et al. Epigenetic inactivation of the Sotos overgrowth syndrome gene histone methyltransferase NSD1 in human neuroblastoma and glioma. Proc Natl Acad Sci USA 2009; 106: 21830–21835. 20018718 10.1073/pnas.0906831106 PMC2793312

[bib10] Weinstein JN, Lorenzi PL. Cancer: discrepancies in drug sensitivity. Nature 2013; 504: 381–383. 24284624 10.1038/nature12839

[bib11] Mendes-Pereira AM, Sims D, Dexter T, Fenwick K, Assiotis I, Kozarewa I et al. Genome-wide functional screen identifies a compendium of genes affecting sensitivity to tamoxifen. Proc Natl Acad Sci USA 2012; 109: 2730–2735. 21482774 10.1073/pnas.1018872108 PMC3286962

[bib12] Wang GG, Cai L, Pasillas MP, Kamps MP. NUP98-NSD1 links H3K36 methylation to Hox-A gene activation and leukaemogenesis. Nat Cell Biol 2007; 9: 804–812. 17589499 10.1038/ncb1608

[bib13] Kuo AJ, Cheung P, Chen K, Zee BM, Kioi M, Lauring J et al. NSD2 links dimethylation of histone H3 at lysine 36 to oncogenic programming. Mol Cell 2011; 44: 609–620. 22099308 10.1016/j.molcel.2011.08.042 PMC3222870

[bib14] Gerlinger M, Rowan AJ, Horswell S, Larkin J, Endesfelder D, Gronroos E et al. Intratumor heterogeneity and branched evolution revealed by multiregion sequencing. N Engl J Med 2012; 366: 883–892. 22397650 10.1056/NEJMoa1113205 PMC4878653

[bib15] Yuan W, Xu M, Huang C, Liu N, Chen S, Zhu B. H3K36 methylation antagonizes PRC2-mediated H3K27 methylation. J Biol Chem 2011; 286: 7983–7989. 21239496 10.1074/jbc.M110.194027 PMC3048685

[bib16] Fraga MF, Ballestar E, Villar-Garea A, Boix-Chornet M, Espada J, Schotta G et al. Loss of acetylation at Lys16 and trimethylation at Lys20 of histone H4 is a common hallmark of human cancer. Nat Genet 2005; 37: 391–400. 15765097 10.1038/ng1531

[bib17] Schotta G, Lachner M, Sarma K, Ebert A, Sengupta R, Reuter G et al. A silencing pathway to induce H3-K9 and H4-K20 trimethylation at constitutive heterochromatin. Genes Dev 2004; 18: 1251–1262. 15145825 10.1101/gad.300704 PMC420351

[bib18] Stender JD, Pascual G, Liu W, Kaikkonen MU, Do K, Spann NJ et al. Control of proinflammatory gene programs by regulated trimethylation and demethylation of histone H4K20. Mol Cell 2012; 48: 28–38. 22921934 10.1016/j.molcel.2012.07.020 PMC3472359

[bib19] Kouzarides T. Chromatin modifications and their function. Cell 2007; 128: 693–705. 17320507 10.1016/j.cell.2007.02.005

[bib20] Benetti R, Gonzalo S, Jaco I, Schotta G, Klatt P, Jenuwein T et al. Suv4-20h deficiency results in telomere elongation and derepression of telomere recombination. J Cell Biol 2007; 178: 925–936. 17846168 10.1083/jcb.200703081 PMC2064618

[bib21] Karin M, Cao Y, Greten FR, Li ZW. NF-kappaB in cancer: from innocent bystander to major culprit. Nat Rev Cancer 2002; 2: 301–310. 12001991 10.1038/nrc780

[bib22] De Santa F, Totaro MG, Prosperini E, Notarbartolo S, Testa G, Natoli G. The histone H3 lysine-27 demethylase Jmjd3 links inflammation to inhibition of polycomb-mediated gene silencing. Cell 2007; 130: 1083–1094. 17825402 10.1016/j.cell.2007.08.019

[bib23] Gopal YN, Arora TS, Van Dyke MW. Parthenolide specifically depletes histone deacetylase 1 protein and induces cell death through ataxia telangiectasia mutated. Chem Biol 2007; 14: 813–823. 17656318 10.1016/j.chembiol.2007.06.007

[bib24] Liu Z, Liu S, Xie Z, Pavlovicz RE, Wu J, Chen P et al. Modulation of DNA methylation by a sesquiterpene lactone parthenolide. J Pharmacol Exp Ther 2009; 329: 505–514. 19201992 10.1124/jpet.108.147934 PMC2672871

[bib25] Shanmugam R, Kusumanchi P, Appaiah H, Cheng L, Crooks P, Neelakantan S et al. A water soluble parthenolide analog suppresses *in vivo* tumor growth of two tobacco-associated cancers, lung and bladder cancer, by targeting NF-kappaB and generating reactive oxygen species. Int J Cancer 2011; 128: 2481–2494. 20669221 10.1002/ijc.25587 PMC2982935

[bib26] Price BD, D'Andrea AD. Chromatin remodeling at DNA double-strand breaks. Cell 2013; 152: 1344–1354. 23498941 10.1016/j.cell.2013.02.011 PMC3670600

[bib27] Modak R, Das Mitra S, Krishnamoorthy P, Bhat A, Banerjee A, Gowsica BR et al. Histone H3K14 and H4K8 hyperacetylation is associated with Escherichia coli-induced mastitis in mice. Epigenetics 2012; 7: 492–501. 22419123 10.4161/epi.19742

[bib28] Patel NM, Nozaki S, Shortle NH, Bhat-Nakshatri P, Newton TR, Rice S et al. Paclitaxel sensitivity of breast cancer cells with constitutively active NF-kappaB is enhanced by IkappaBalpha super-repressor and parthenolide. Oncogene 2000; 19: 4159–4169. 10962577 10.1038/sj.onc.1203768

[bib29] Hoffmann A, Leung TH, Baltimore D. Genetic analysis of NF-kappaB/Rel transcription factors defines functional specificities. EMBO J 2003; 22: 5530–5539. 14532125 10.1093/emboj/cdg534 PMC213788

[bib30] Nozaki S, Sledge GW Jr, Nakshatri H. Repression of GADD153/CHOP by NF-kappaB: a possible cellular defense against endoplasmic reticulum stress-induced cell death. Oncogene 2001; 20: 2178–2185. 11360202 10.1038/sj.onc.1204292

[bib31] Mott JL, Kurita S, Cazanave SC, Bronk SF, Werneburg NW, Fernandez-Zapico ME. Transcriptional suppression of mir-29b-1/mir-29a promoter by c-Myc, hedgehog, and NF-kappaB. J Cell Biochem 2010; 110: 1155–1164. 20564213 10.1002/jcb.22630 PMC2922950

[bib32] Lin YC, Hsu EC, Ting LP. Repression of hepatitis B viral gene expression by transcription factor nuclear factor-kappaB. Cell Microbiol 2009; 11: 645–660. 19141126 10.1111/j.1462-5822.2008.01280.x

[bib33] Dhawan P, Richmond A. Role of CXCL1 in tumorigenesis of melanoma. J Leukoc Biol 2002; 72: 9–18. 12101257 PMC2668262

[bib34] Chua HL, Bhat-Nakshatri P, Clare SE, Morimiya A, Badve S, Nakshatri H. NF-kappaB represses E-cadherin expression and enhances epithelial to mesenchymal transition of mammary epithelial cells: potential involvement of ZEB-1 and ZEB-2. Oncogene 2007; 26: 711–724. 16862183 10.1038/sj.onc.1209808

[bib35] Kapoor-Vazirani P, Kagey JD, Vertino PM. SUV420H2-mediated H4K20 trimethylation enforces RNA polymerase II promoter-proximal pausing by blocking hMOF-dependent H4K16 acetylation. Mol Cell Biol 2011; 31: 1594–1609. 21321083 10.1128/MCB.00524-10 PMC3126334

[bib36] Kooistra SM, Helin K. Molecular mechanisms and potential functions of histone demethylases. Nat Rev Mol Cell Biol 2012; 13: 297–311. 22473470 10.1038/nrm3327

[bib37] Lu T, Jackson MW, Singhi AD, Kandel ES, Yang M, Zhang Y et al. Validation-based insertional mutagenesis identifies lysine demethylase FBXL11 as a negative regulator of NFkappaB. Proc Natl Acad Sci USA 2009; 106: 16339–16344. 19805303 10.1073/pnas.0908560106 PMC2736141

[bib38] Ge R, Wang Z, Zeng Q, Xu X, Olumi AF. F-box protein 10, an NF-kappaB-dependent anti-apoptotic protein, regulates TRAIL-induced apoptosis through modulating c-Fos/c-FLIP pathway. Cell Death Differ 2011; 18: 1184–1195. 21252908 10.1038/cdd.2010.185 PMC3131965

[bib39] Nakshatri H, Rice SE, Bhat-Nakshatri P. Antitumor agent parthenolide reverses resistance of breast cancer cells to tumor necrosis factor-related apoptosis-inducing ligand through sustained activation of c-Jun N-terminal kinase. Oncogene 2004; 23: 7330–7344. 15286701 10.1038/sj.onc.1207995

[bib40] Gao J, Aksoy BA, Dogrusoz U, Dresdner G, Gross B, Sumer SO et al. Integrative analysis of complex cancer genomics and clinical profiles using the cBioPortal. Sci Signal 2013; 6: pl1. 23550210 10.1126/scisignal.2004088 PMC4160307

[bib41] Cerami E, Gao J, Dogrusoz U, Gross BE, Sumer SO, Aksoy BA et al. The cBio cancer genomics portal: an open platform for exploring multidimensional cancer genomics data. Cancer Discov 2012; 2: 401–404. 22588877 10.1158/2159-8290.CD-12-0095 PMC3956037

[bib42] Dyrskjot L, Kruhoffer M, Thykjaer T, Marcussen N, Jensen JL, Moller K et al. Gene expression in the urinary bladder: a common carcinoma *in situ* gene expression signature exists disregarding histopathological classification. Cancer Res 2004; 64: 4040–4048. 15173019 10.1158/0008-5472.CAN-03-3620

[bib43] Goswami CP, Nakshatri H. PROGgene: gene expression based survival analysis web application for multiple cancers. J Clin Bioinformatics 2013; 3: 22. 10.1186/2043-9113-3-22 PMC3875898 24165311

[bib44] Gyorffy B, Lanczky A, Eklund AC, Denkert C, Budczies J, Li Q et al. An online survival analysis tool to rapidly assess the effect of 22,277 genes on breast cancer prognosis using microarray data of 1,809 patients. Breast Cancer Res Treat 2010; 123: 725–731. 20020197 10.1007/s10549-009-0674-9

[bib45] Nakshatri H, Bhat-Nakshatri P, Martin DA, Goulet RJ Jr, Sledge GW Jr. Constitutive activation of NF-kappaB during progression of breast cancer to hormone-independent growth. Mol Cell Biol 1997; 17: 3629–3639. 9199297 10.1128/mcb.17.7.3629 PMC232215

[bib46] Zhou Y, Eppenberger-Castori S, Eppenberger U, Benz CC. The NFkappaB pathway and endocrine-resistant breast cancer. Endocr Relat Cancer 2005; 12: S37–S46. 16113098 10.1677/erc.1.00977

[bib47] Riggins RB, Zwart A, Nehra R, Clarke R. The nuclear factor kappa B inhibitor parthenolide restores ICI 182,780 (Faslodex; fulvestrant)-induced apoptosis in antiestrogen-resistant breast cancer cells. Mol Cancer Ther 2005; 4: 33–41. 15657351

[bib48] Czabotar PE, Lessene G, Strasser A, Adams JM. Control of apoptosis by the BCL-2 protein family: implications for physiology and therapy. Nat Rev Mol Cell Biol 2013; 15: 49–63. 10.1038/nrm3722 24355989

[bib49] Huang N, vom Baur E, Garnier JM, Lerouge T, Vonesch JL, Lutz Y et al. Two distinct nuclear receptor interaction domains in NSD1, a novel SET protein that exhibits characteristics of both corepressors and coactivators. EMBO J 1998; 17: 3398–3412. 9628876 10.1093/emboj/17.12.3398 PMC1170677

[bib50] Stephens PJ, Tarpey PS, Davies H, Van Loo P, Greenman C, Wedge DC et al. The landscape of cancer genes and mutational processes in breast cancer. Nature 2012; 486: 400–404. 22722201 10.1038/nature11017 PMC3428862

[bib51] Xie P, Tian C, An L, Nie J, Lu K, Xing G et al. Histone methyltransferase protein SETD2 interacts with p53 and selectively regulates its downstream genes. Cell Signal 2008; 20: 1671–1678. 18585004 10.1016/j.cellsig.2008.05.012

[bib52] Dong J, Jimi E, Zhong H, Hayden MS, Ghosh S. Repression of gene expression by unphosphorylated NF-kappaB p65 through epigenetic mechanisms. Genes Dev 2008; 22: 1159–1173. 18408078 10.1101/gad.1657408 PMC2335313

[bib53] Beauchef G, Bigot N, Kypriotou M, Renard E, Poree B, Widom R et al. The p65 subunit of NF-kappaB inhibits COL1A1 gene transcription in human dermal and scleroderma fibroblasts through its recruitment on promoter by protein interaction with transcriptional activators (c-Krox, Sp1, and Sp3). J Biol Chem 2012; 287: 3462–3478. 22139845 10.1074/jbc.M111.286443 PMC3271000

[bib54] Beg AA, Baltimore D. An essential role for NF-kappaB in preventing TNF-alpha-induced cell death [see comments]. Science 1996; 274: 782–784. 8864118 10.1126/science.274.5288.782

[bib55] Koboldt DC, Fulton RS, McLellan MD, Schmidt H, Kalicki-Veizer J, McMichael JF et al. Comprehensive molecular portraits of human breast tumours. Nature 2012; 490: 61–70. 23000897 10.1038/nature11412 PMC3465532

