# Supplementary material for: NF-κB-dependent and -independent epigenetic modulation using the novel anti-cancer agent DMAPT
Source: Cell Death Dis. 2015 Jan 22;6(1):e1608–. doi: 10.1038/cddis.2014.569 (PMC4669767; doi:10.1038/cddis.2014.569)
Supplement: Supplementary Table S1 [file cddis2014569x1.docx]

| **Table S1: List of qRT-PCR primers** | | |
| --- | --- | --- |
| **Gene** | **qPCR Forward primer** | **qPCR Reverse primer** |
| Human NSD1 | 5’ TTC AGG AAG CTG CAG CAA GGT TTG 3’ | 5’ TGT ACC CTG CCA ATA GGA CGG TTT 3’ |
| Mouse NSD1 | 5’ TTG TCC TTG GAG GAG CAT CTG GTT 3’ | 5’ TTG CCA ATC GCC CTT GTT CAC TTC 3’ |
| Human SETD2 | 5’ ACT CTC CTG CAC AGT CTC AAG CAA 3’ | 5’ AGA CTT GGC TGG GCA TAA CTC TGT 3’ |
| Mouse SETD2 | 5’ TCT CCC AAA TAA GTC TCG GCA GCA 3’ | 5’ GCA AGC CAT CCA TTC GGG AAA TGT 3’ |
| Human β-actin | 5’ AAT GAG GCC GAG GAC TTT GAT TGC 3’ | 5’ AGG ATG GCA AGG GAC TTC CTG TAA 3’ |
| Mouse β-actin | 5’ TGT GAT GGT GGG AAT GGG TCA GAA 3’ | 5’ TGT GGT GCC AGA TCT TCT CCA TGT 3’ |
| Human KDM2B | 5’ TCT GCC AAA TGG ACC CAT CTC ACT 3’ | 5’ TGC TCC TTC AGG ACG TTC TTC ACA 3’ |
| Mouse KDM2B | 5’ TGA CCT GTG CCT GTC CTT CTT CAA 3’ | 5’ AGG AGT TTC TCT TCC ACT TGC CCA 3’ |
| Human KDM4A | 5’ TGA GTT TGA AGA GCT CGA GCG GAA 3’ | 5’ AGG GTA CCA TTC ACA TCT GCA CCA 3’ |
| Human KDM4B | 5’ CTG CGC CCA CCT TTG CAG AAT AAA 3’ | 5’ TCT GGA ACA CCT GCG TTT CTC TCA 3’ |
| Human KMT5C | 5’ ACC CAA CTG CAA GTT TGT GCC T 3’ | 5’ TCA CAG GTG TGG CAT TCA CAG T 3’ |
| Mouse KMT5C | 5’ AAC CAT GAC TGC AAA CCC AAC TGC 3’ | 5’ TCA ATG TCC CGT AGC ACC TTC ACA 3’ |
| Human CXCL1 | 5’ ATG GCC CGC GCT CTC TCC 3’ | 5’ CAG GAA CAG CCA CCA GTG AGC 3’ |
