# Supplementary figures and images for: NF-κB-dependent and -independent epigenetic modulation using the novel anti-cancer agent DMAPT
Source: Cell Death Dis. 2015 Jan 22;6(1):e1608–. doi: 10.1038/cddis.2014.569 (PMC4669767; doi:10.1038/cddis.2014.569)

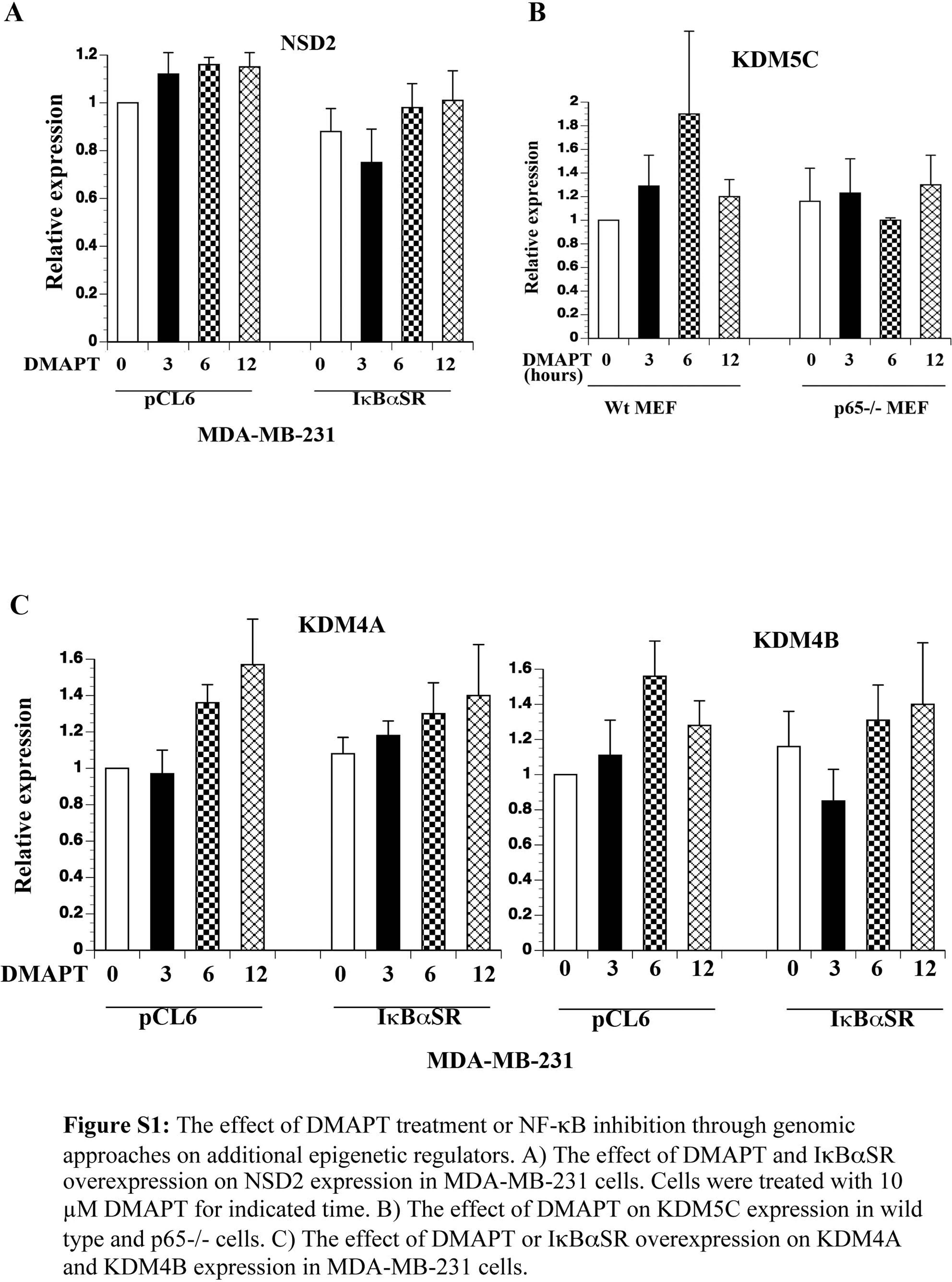

Supplement: Supplementary Figure S1 [file cddis2014569x2.tif]

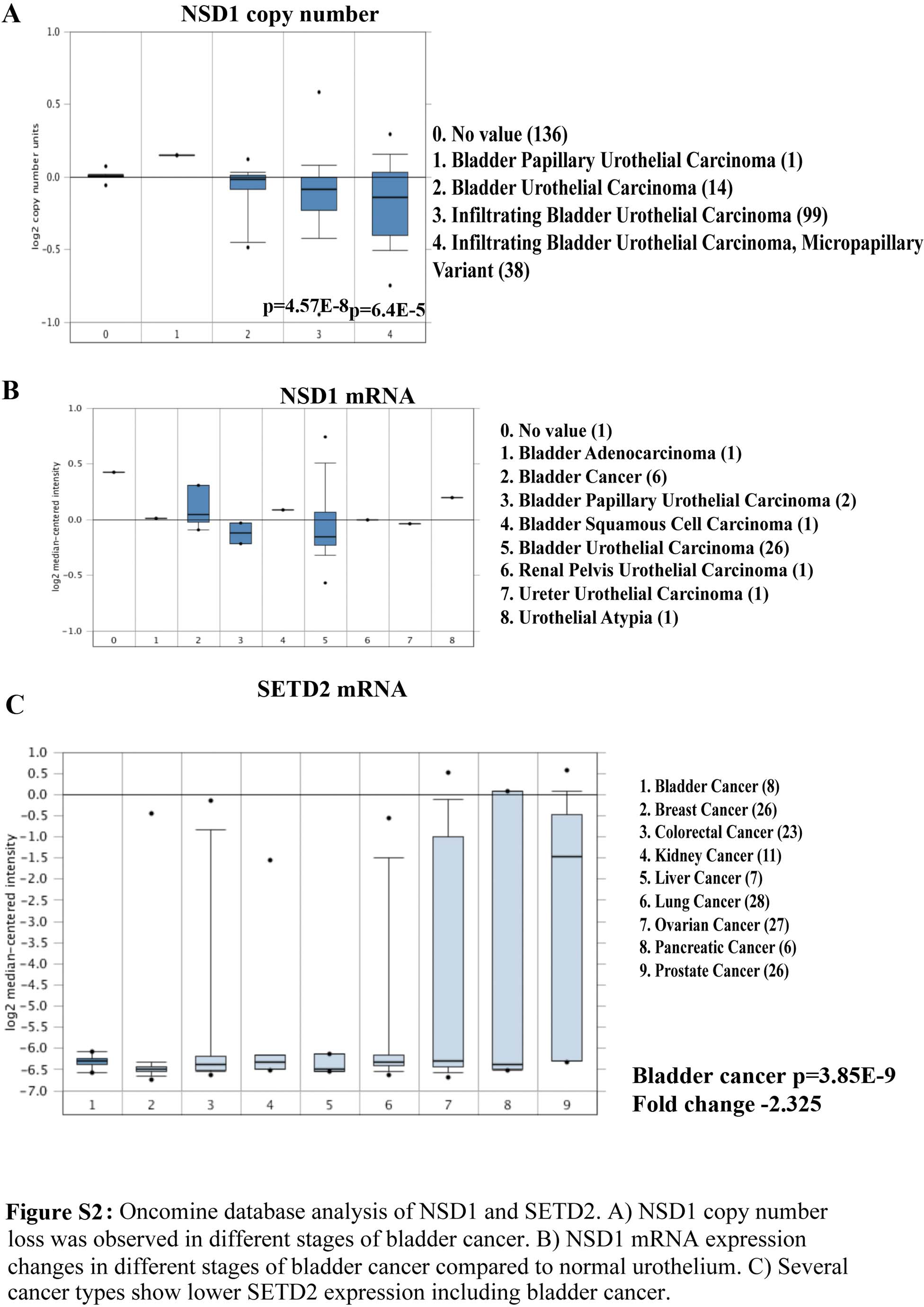

Supplement: Supplementary Figure S2 [file cddis2014569x3.tif]
